# Immature Follicular Origins and Disrupted Oocyte Growth Pathways Contribute to Decreased Gamete Quality During Reproductive Juvenescence in Mice

**DOI:** 10.3389/fcell.2021.693742

**Published:** 2021-06-16

**Authors:** Atsuko Kusuhara, Elnur Babayev, Luhan T. Zhou, Vijay P. Singh, Jennifer L. Gerton, Francesca E. Duncan

**Affiliations:** ^1^Department of Obstetrics and Gynecology, Feinberg School of Medicine, Northwestern University, Chicago, IL, United States; ^2^Stowers Institute for Medical Research, Kansas City, MO, United States

**Keywords:** aging, puberty, follicle, meiosis, zona pellucida, cumulus cells, oocyte, spindle

## Abstract

Egg quality dictates fertility outcomes, and although there is a well-documented decline with advanced reproductive age, how it changes during puberty is less understood. Such knowledge is critical, since advances in Assisted Reproductive Technologies are enabling pre- and peri-pubertal patients to preserve fertility in the medical setting. Therefore, we investigated egg quality parameters in a mouse model of the pubertal transition or juvenescence (postnatal day; PND 11–40). Animal weight, vaginal opening, serum inhibin B levels, oocyte yield, oocyte diameter, and zona pellucida thickness increased with age. After PND 15, there was an age-associated ability of oocytes to resume meiosis and reach metaphase of meiosis II (MII) following *in vitro* maturation (IVM). However, eggs from the younger cohort (PND 16–20) had significantly more chromosome configuration abnormalities relative to the older cohorts and many were at telophase I instead of MII, indicative of a cell cycle delay. Oocytes from the youngest mouse cohorts originated from the smallest antral follicles with the fewest cumulus layers per oocyte, suggesting a more developmentally immature state. RNA Seq analysis of oocytes from mice at distinct ages revealed that the genes involved in cellular growth signaling pathways (PI3K, mTOR, and Hippo) were consistently repressed with meiotic competence, whereas genes involved in cellular communication were upregulated in oocytes with age. Taken together, these data demonstrate that gametes harvested during the pubertal transition have low meiotic maturation potential and derive from immature follicular origins.

## Introduction

Female reproductive aging is associated with a striking reduction in gamete quantity and quality beginning when women reach their mid-thirties, and this contributes to both loss of fertility and endocrine function (Hassold and Chiu, 1985; Speroff, 1994; Armstrong, 2001; Franasiak et al., 2014). Much less is known about how egg quality is impacted at the other end of the age spectrum because pregnancy in adolescence is avoided in most cultures and societies. However, this need is rapidly emerging in the medical and particularly pediatric oncology setting. Due to clinical advances in diagnostics and therapeutics, more than 80% of children with a malignancy will achieve a 5-year survival, making approximately 1 in 750 a childhood cancer survivor in the United States (Ward et al., 2014). Despite these life-saving improvements, childhood cancer survivors show accelerated aging phenotypes relative to their cancer-free siblings (Green et al., 2009). With regards to reproductive function, survivors are more likely than their siblings to experience infertility (Green et al., 2009; Nielsen et al., 2013). This has fueled the field of Oncofertility—the merging of oncology and fertility—to explore and expand fertility preservation options for men, women, and children (Gracia and Woodruff, 2012; Woodruff, 2015).

For females, standard fertility preservation options include oocyte and embryo banking, but these techniques are not suitable for prepubertal girls. In these populations, technologies such as ovarian tissue cryopreservation (OTC), as well as *ex vivo in vitro* maturation (IVM) can be used (Anderson and Wallace, 2011; Wang et al., 2016; Algarroba et al., 2018). In OTC, ovarian tissue is removed from the patient and cortical strips containing primordial follicles are cryopreserved to later be used for transplantation to restore endocrine function and/or fertility. While ovarian tissue transplantation has resulted in >130 live births worldwide, the knowledge of its success using tissue isolated from prepubertal and adolescent girls is limited but is increasing as the individuals who have stored ovarian tissue are surviving their cancer and reaching the age where they want to restore their reproductive potential (Duncan et al., 2015; Jensen et al., 2017a,b; Takae et al., 2019). *Ex vivo* IVM is an emerging method that can be coupled with OTC to broaden the fertility preservation options for young girls (Revel et al., 2009; Anderson and Wallace, 2011). In this method, immature oocytes are collected *ex vivo* from small antral follicles at the same time that the harvested ovarian tissue is prepared into cortical strips for OTC (Kristensen et al., 2011). These oocytes are then matured *in vitro*, and mature eggs arrested at metaphase of meiosis II (MII) are cryopreserved for the female’s future use (Revel et al., 2009). Fertilization of eggs derived from *ex vivo* IVM has resulted in pregnancies and live birth, and mature eggs have been obtained successfully from premenarchal and adolescent girls alongside frozen ovarian tissue (Revel et al., 2003; Uzelac et al., 2015; Fasano et al., 2017). The use of such methods in young females is increasing, and in the past 5 years more than 70% of the participants who have undergone OTC through the Oncofertility Consortium’s National Physicians Cooperative were under the age of 18 (Duncan et al., 2015; Armstrong et al., 2018).

Given these trends in fertility preservation, the eggs that normally would not have contributed to physiologic fertility will in fact be used clinically. Thus, there is a critical need to examine egg quality parameters in young females. In fact, compelling evidence already exists that egg quality may be suboptimal at the youngest ages in addition to advanced reproductive age. Many studies show inferior developmental capacity of oocytes obtained from younger, pre-pubertal mammals (Pinkert et al., 1989; Eppig et al., 1992; Revel et al., 1995; Damiani et al., 1996; Khatir et al., 1996; O’Brien et al., 1996, 1997; Ledda et al., 1997; Izquierdo et al., 1999). In addition, girls experience a one to 3 year period of adolescent sterility or subfecundity following menarche, and this phenomenon is evolutionarily conserved (Hartman, 1931). Moreover, the incidence of egg aneuploidy and trisomies in clinically recognized pregnancies exhibit a u- or j-shaped curve, respectively, where the frequency of these events is the highest at both ends of the reproductive spectrum (Hassold and Chiu, 1985; Gruhn et al., 2019). This pattern was also observed in a comprehensive chromosomal screening study of human embryos demonstrating that the incidence of aneuploidy was >40% for women in the early twenties, compared to 20–25% in mid to late twenties (Franasiak et al., 2014). Puberty involves a complex series of maturational events involving the development of the hypothalamic-pituitary-ovarian axis that drives oogenesis and folliculogenesis. Therefore, we hypothesized that puberty is a particularly sensitive window in the development of a high quality gamete and investigated the determinants of egg quality using a mouse model of the pubertal transition.

## Results

### Characterization of a Mouse Model of the Pubertal Transition

**FIGURE 1:**
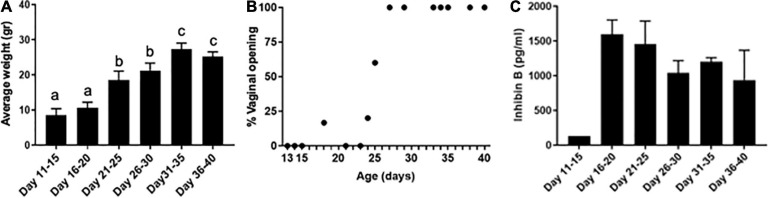
Pubertal transition in mice as characterized by changes in weight, vaginal opening, and inhibin B levels. **(A)** Average mouse weight in different age cohorts was measured across pubertal transition (*n* = 42, 5—9 mice per age cohort). Error bars represent standard error of the mean (SEM). Columns with different letterheads are significantly different from each other (*p* < 0.001). **(B)** Percent of mice with vaginal opening was tracked at different ages spanning pubertal transition (*n* = 39). Mice were not tracked longitudinally; instead, mice within each age cohort represented distinct biological replicates and were euthanized immediately following the evaluation of vaginal opening. **(C)** Serum inhibin B levels were measured in mice across pubertal transition (*n* = 21 mice, 3–5 mice per age cohort—one pooled serum sample from 5 mice was tested for PND 11–15, and individual serum samples were used in other age groups).

We used a mouse model of the pubertal transition and an experimental paradigm that mimics how gametes are obtained clinically in the fertility preservation setting for young females, whereby oocytes from antral follicles are isolated from unstimulated ovaries and matured *in vitro* to obtain eggs arrested at metaphase stage of meiosis II (MII) (Revel et al., 2009; Abir et al., 2016). In mice, puberty typically occurs more than 20 days after birth (Vandenbergh, 1967; Smitz and Cortvrindt, 2002). Increased gonadotropin-releasing hormone (GnRH) pulse frequency is an important event that marks puberty. In rodents, GnRH is secreted in a pulsatile fashion beginning at PND 5, and the highest GnRH pulse frequencies are observed between PND 40–45 (Prevot et al., 2003). Therefore, we examined gamete quality endpoints in age cohorts of mice spanning puberty: PND 11–15, 16–20, 21–25, 26–30, 31–35, and 36–40. To confirm that these age cohorts spanned this period of development, we examined animal weights and vaginal opening. Growth patterns are an important factor in assessing pubertal timing, and weight increase is associated with puberty (Kennedy and Mitra, 1963). Animal weight increased significantly over the pubertal transition from 8.6 ± 1.8 g on PND 11–15 to 25.3 ± 1.3 g by PND 36–40 (*p* < 0.001, Figure 1A). Vaginal opening is often used as an outward manifestation of puberty (Caligioni, 2009). In fact, significantly fewer GnRH pulse frequencies are observed in animals prior to vaginal opening compared to those that are analyzed after vaginal opening, suggesting a tight link between vaginal opening and pubertal status (Sisk et al., 2001). In our model, vaginal opening was first observed in a subset of mice beginning on PND 18 (Figure 1B). The percentage of mice exhibiting vaginal opening increased until PND 27, after which point 100% of animals were positive for this phenotype, suggesting that they had reached puberty (Figure 1B).

To further characterize that our age cohorts spanned the pubertal transition, we analyzed serum levels of the gonadal peptide inhibin B (Figure 1C). Serum inhibin B levels are a marker of follicular activity which occurs during the pubertal transition, including an increased number of developing follicles and those reaching a later stage of development (Rivier and Vale, 1987; Raivio and Dunkel, 2002). In our model, inhibin B levels were low in PND 11–15 mice (127.3 pg/ml) but increased dramatically in the PND 16–20 cohort (1,595.25 ± 179.32 pg/ml), suggesting that these time points delineate an important transition in the onset of puberty (Figure 1C). We also measured estradiol levels and found that they were detectable across the age cohorts. The levels were less than 10 pg/ml and not significantly different across age groups. However, there was a trend of increasing estradiol levels from 3.8 ± 0.5 pg/ml in PND 16–20 mice to 8.2 ± 1.1 pg/ml in PND 26–30 mice.

### Oocyte Diameter and Meiotic Competence Increases Across the Pubertal Transition

**FIGURE 2:**
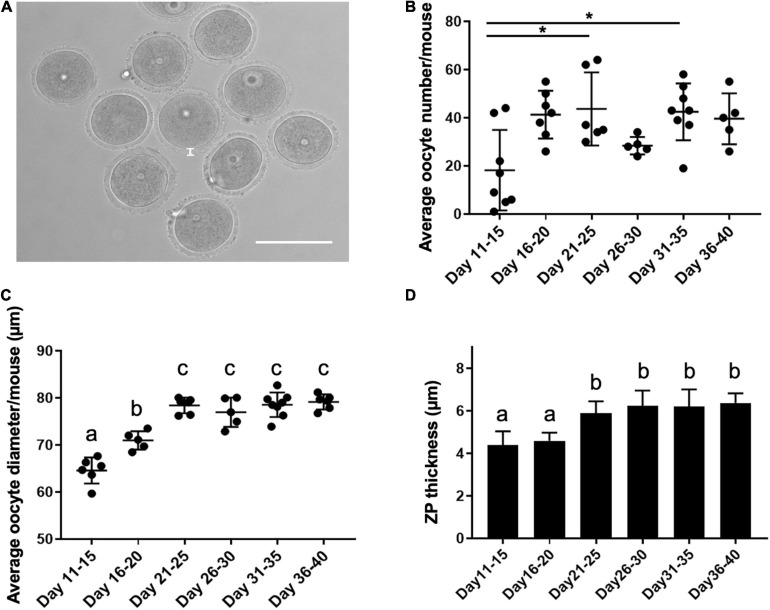
Oocyte characteristics during the pubertal transition as assessed by yield, diameter, and zona pellucida thickness. **(A)** A representative transmitted light image of oocytes isolated from antral follicles. The thickness of the zona pellucida (ZP) is delineated by the white line. The scale bar is 100 μm. **(B)** The average number of oocytes collected per mouse in each age cohort was examined (*n* = 36 mice, 5–8 mice per age cohort). Asterisks denote groups significantly different from each other (*p* < 0.01). **(C)** The average oocyte diameter was calculated for oocytes collected from each mouse in distinct age cohorts (*n* = 363 oocytes, 36 mice, 4–8 mice per age cohort). Groups with different letterheads are significantly different from each other (*p* < 0.01). **(D)** Zona pellucida thickness was measured for a total of 113 oocytes from 7 to 15 mice per age cohort, and the average values are plotted. Error bars represent standard deviation (SD). Column bars with different letterheads are significantly different from each other (*p* < 0.01).

To determine how the pubertal transition affected oocyte characteristics, we counted the average number of oocytes collected per mouse and measured oocyte diameter and zona pellucida thickness across the age cohorts (Figure 2A). Although the pattern was not consistent across all cohorts, there did appear to be a general increase in oocyte yield with increasing age (Figure 2B). For example, relative to PND 11–15, significantly more oocytes were collected at PND 21–25 and PND 31–35 (18.3 ± 16.7 oocytes/mouse, 43.7 ± 15.2 oocytes/mouse, and 42.5 ± 11.8 oocytes/mouse, respectively, *p* < 0.01). The ratio of oocytes enclosed in cumulus oocyte complexes to denuded ones upon isolation was similar across cohorts (Supplementary Figure 1). There was a clear age-associated increase in oocyte diameter (Figure 2C). Average oocyte diameter increased significantly from 65.6 ± 4.7 μm on PND 11–15 to 71.0 ± 4.1 μm on PND 16–20 and 78.4 ± 4.6 μm on PND 21–25 (*p* < 0.01, Figure 2C). Oocytes reached maximal diameter on PND 21–25, after which point there was no further increase. To further assess how oogenesis may differ across the pubertal transition, we examined the average thickness of the zona pellucida in oocytes collected from mice in different age cohorts (Figure 2D). The zona pellucida is a glycoprotein matrix secreted by the oocyte during its growth phase, and positive correlations between zona thickness and maturation, fertilization, and preimplantation embryo development outcomes have been reported in multiple species (Conner et al., 2005). Zona pellucida thickness increased significantly from 4.4 ± 0.5 μm in oocytes from PND 11–15 mice to approximately 6.0 μm in the cohorts beyond PND 21 (Figure 2D, *p* < 0.01).

**FIGURE 3:**
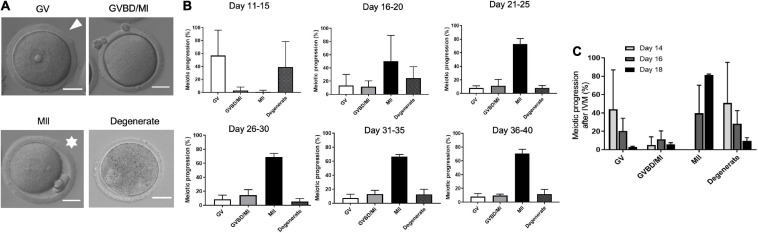
Age-dependent acquisition of oocyte meiotic competence in mice. **(A)** The ability of the oocytes in each age cohort to progress through meiosis following *in vitro* maturation (IVM) was scored according to morphological criteria as visible in the representative transmitted light images. Cells with an intact germinal vesicle (GV; arrowhead) were arrested at prophase I; cells that had extruded a first polar body (asterisks) reached metaphase of meiosis II (MII); cells that lacked a GV or a PB had undergone germinal vesicle breakdown (GVBD) or were at metaphase of meiosis I (MI). Fragmented, lysed, or shrunken cells were classified as degenerated. The scale bars are 25 μm. **(B)** Percentage of oocytes isolated from mice that progressed to specific stages of meiosis following IVM was calculated for each age cohort (*n* = 34 mice, 4–8 mice per age cohort). **(C)** Percentage of oocytes isolated from PND 14, PND 16, and PND 18 mice that progressed to specific stages of meiosis following IVM. MII-arrested eggs are first observed on PND 16. PND, postnatal day.

A key hallmark of gamete quality is the acquisition of meiotic competence or the ability of the oocyte to resume meiosis and reach MII at which stage it can be fertilized. To define the precise time when oocytes achieve meiotic competence, we performed IVM and scored the ability of oocytes isolated from the different age cohorts to progress through meiosis (Figure 3A). Oocytes isolated from mice in the PND 11–15 cohort lacked meiotic competence, with only 1.0 ± 2.5% of the cells reaching MII (Figure 3B and Supplementary Figure 2C). In fact, this age cohort had a significantly higher percentage of cells (54.2 ± 38.5%, *p* < 0.05) arrested in prophase I (GV-intact) relative to the other age cohorts (Figure 3B and Supplementary Figure 2A). After PND 15, the ability of oocytes to reach MII following IVM increased to between 55.4 and 72.7% in the older age cohorts (Figure 3B and Supplementary Figure 2C). Although there was no significant difference in meiotic competence among the age cohorts after PND 15, the PND16–20 cohort exhibited the largest variation (Supplementary Figure 2C). Therefore, we examined meiotic competence more closely over 2-day intervals during this early period of the pubertal transition. Meiotic competence increased steadily over this period, with 0% of cells reaching MII on PND 14, 39.7 ± 30.5% of cells on PND 16, and 81.5 ± 0.9% on PND 18 (Figure 3C). Interestingly, we noted that PND 16 mice naturally segregated into two distinct subgroups by weight, and the heavier group (≥9 g) had more meiotically competent oocytes >50% compared to <10% in mice that weighed <9 g (data not shown).

### Altered Chromosome Configurations Following IVM Are Highest in Eggs Obtained From Mice During Pubertal Transition Period

**FIGURE 4:**
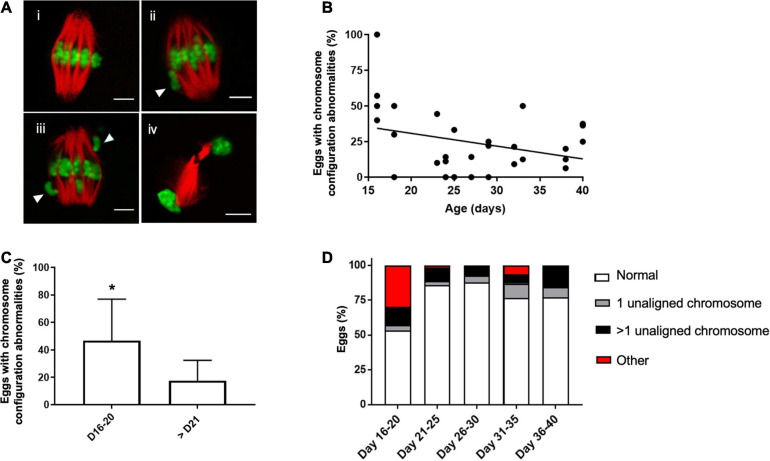
Abnormal meiotic chromosome configurations are more prevalent in the youngest age cohort. **(A)** Representative confocal images of how different chromosome configurations observed in MII eggs were categorized: (i) normal bipolar spindle with chromosomes aligned on the metaphase plate (ii) spindle with 1 misaligned chromosome (arrowhead), (iii) spindle with (>1 misaligned chromosomes (arrowheads), and (iv) a telophase I spindle indicative of cell cycle delay. The scale bar is 5 μm. **(B)** The correlation between mouse age and the percentage of eggs with abnormal chromosome configurations (*n* = 244 eggs from 4 to 8 mice per age cohort). Line was fit to the data (*r* = −0.22, *p* = 0.23). **(C)** The average incidence of chromosomal abnormalities in MII eggs per mouse in the PND 16–20 was plotted relative to the other ages in aggregate. Asterisk denotes significant difference between the groups (*p* < 0.01, *n* = 244 eggs from 4 to 8 mice per age cohort). **(D)** Percentage of MII eggs with specific chromosome configuration abnormalities was plotted in each age cohort according to the criteria established in **(A)**.

Although the majority of cells in mice > PND 15 were able to reach MII following IVM, the quality of the resulting gametes may not be equivalent across the pubertal transition. Chromosome segregation is essential for oocyte meiosis and defects in this process can result in aneuploidy. Therefore, we examined whether chromosome configuration defects were observed in mature eggs across the pubertal transition. To do this, we divided the spindle phenotypes observed in MII eggs into four main categories: (i) normal chromosome alignment along the metaphase plate, (ii) 1 chromosome misaligned on the metaphase plate, (iii) >1 chromosome misaligned on the metaphase plate, and (iv) other configurations such as telophase I (Figure 4A). We observed an inverse relationship between the age of mice (PND 15–40) and percentage of eggs with chromosome configuration abnormalities (Figure 4B). Although this relationship was weak and not statistically significant (*r* = −0.22, *p* = 0.23), the incidence of overall chromosome abnormalities was significantly higher in the PND 16–20 cohort when compared to the older age cohorts (46.7 ± 30.2% vs. 17.6 ± 14.8%, *p* < 0.01, Figure 4C). We further subdivided the types of abnormalities observed and found that the majority of abnormal cells in the PND 16–20 cohort exhibited telophase I configurations, indicative of a cell cycle delay in meiotic progression (Figure 4D).

### Decreased Egg Quality Parameters Are Associated With Smaller Follicle Diameters and Reduced Cumulus Cell Layers

**FIGURE 5:**
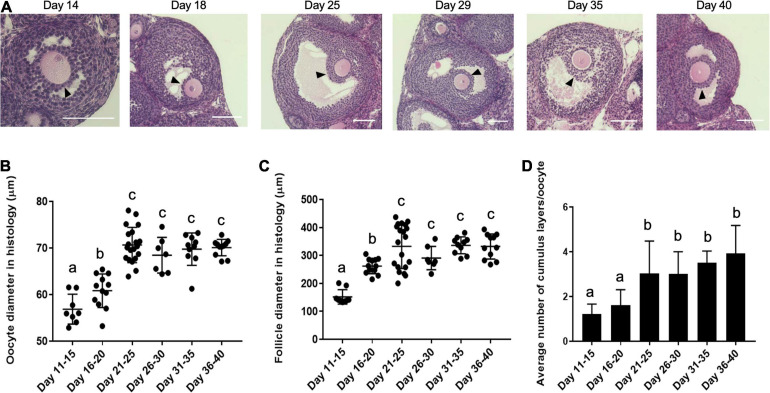
Histological analysis demonstrates that oocytes originate from follicles with increasing diameter and attain more cumulus cell layers as puberty progresses. **(A)** Representative histology sections of antral follicles in mice at different ages across the pubertal transition that contain oocytes of the approximate size as those that were isolated directly from the ovary (Figure 2C). The arrowheads highlight the cumulus cell layers. The scale bars are 100 μm. **(B)** The average oocyte diameter in antral follicles in ovarian histological sections in the different age cohorts are shown (*n* = 68 oocytes within ovaries from at least 2 mice per age cohort analyzed, *p* < 0.01). **(C)** The average follicle diameter of the oocytes measured in **(B)** was plotted (*n* = 68 follicles within ovaries from at least 2 mice per age cohort analyzed, *p* < 0.01). **(D)** The average number of cumulus cell layers surrounding the oocytes in the follicles assessed in **(B,C)** were counted in the section containing the oocyte nucleus as shown in **(A)** (*n* = 68 cumulus-oocyte-complexes within ovaries from at least 2 mice per age cohort analyzed). Cohorts with different letterheads are significantly different from each other (*p* < 0.01).

Coordinated growth between the oocyte and its surrounding somatic cells in the follicle during oogenesis and folliculogenesis is essential for the generation of a high-quality gamete (Hyun et al., 2003; Magalhaes-Padilha et al., 2013). Thus, we wanted to examine whether the differences we observed in egg quality across the pubertal transition could have origins at the level of the follicle. Therefore, we examined histological sections of ovarian tissue from mice in each of the age cohorts to identify follicles that contained oocytes with the approximate diameter as those collected directly from ovarian tissue (Figures 2C, 5A,B). In formalin fixed paraffin embedded tissue (FFPE) ovarian tissue sections, we observed that oocyte diameter significantly increased from 56.9 ± 3.2 μm on PND 11–15 to 60.8 ± 3.6 μm on PND 16–20 and 70.7 ± 3.8 μm on PND 21–25 (*p* < 0.01). Oocytes reached maximal diameter on PND 21–25, after which point there was no further increase with increasing age (Figure 5B). The slight discrepancy in average diameters between freshly isolated oocytes and those observed in FFPE was likely an artifact of tissue fixation and processing. We then measured the corresponding follicle diameter for each oocyte and found that the average follicle diameter increased during the pubertal transition from 152.0 ± 25.8 μm on PND 11–15 to 262.1 ± 27.5 μm on PND 16–20 and 332.7 ± 77.4 μm on PND 21–25 (*p* < 0.01) (Figure 5C). The average follicle diameter plateaued after this point. Thus, oocytes from the youngest age cohorts are derived from small antral follicles which likely accounts for their small size.

At the time of antral cavity formation, the somatic component of the follicle differentiates into two cell types—mural granulosa cells and cumulus cells. The cumulus cells directly surround the oocyte and participate in bidirectional communication to support the oocyte’s metabolic demands (Tanghe et al., 2002; Kordus and LaVoie, 2017). To investigate whether there were differences in the cumulus cell layers that surrounded oocytes across pubertal transition, we counted the average number of layers in each antral follicle that we measured. An average of 1.61 ± 0.68 layers of cumulus cells surrounded oocytes in follicles from the youngest cohort, whereas this gradually increased to 3.92 ± 1.20 layers of cumulus cells per oocyte in follicles from the oldest cohort (*p* < 0.01) (Figure 5D). This histological analysis demonstrates that the increase in oocyte diameter observed across the pubertal transition is associated with a corresponding increase in the average follicle diameter and number of cumulus cell layers surrounding the oocyte. Therefore, the lack of meiotic competence observed in oocytes from PND 11–15 cohort as well as the increase in meiotic defects observed in oocytes from PND 16–20 cohort is likely explained by their origin from small, immature follicles.

### Gene Expression Signatures Differentiate Oocytes Based on Meiotic Competence and Quality

**FIGURE 6:**
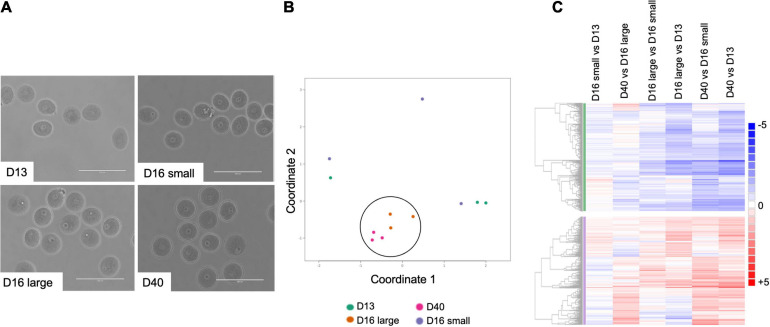
Oocytes collected from mice across the pubertal transition have differential patterns of gene expression. **(A)** Representative transmitted light images of oocytes used for RNASeq analysis from mice at different ages: PND 13, PND 16 small (from small mice), PND 16 large (from large mice), and PND 40. **(B)** Multidimensional scaling (MDS) plot shows oocytes from PND 16 large and PND 40 clustered together (circle) as compared to PND 13 and PND 16 small oocytes. **(C)** Pairwise analysis of differentially expressed genes (fold change +1.5 and −1.5 and adj *p* < 0.05) across different oocyte cohorts shows similar gene expression patterns when comparing PND 16 large with PND 13, PND 40 with PND 16 small and PND 40 with PND 13.

To understand the molecular signatures that underlie meiotic competence and egg quality during the pubertal transition, we performed RNA-Seq on oocytes from PND 13, 16, and PND 40 mice (Figure 6A). The PND 16 animals were further sub-divided into those that weighed less (<9 g) (PND 16 small) and those that weighed more (≥9 g) (PND 16 large). These ages broadly represent mice with oocytes that lack meiotic competence (PND 13, PND 16 small), oocytes that have meiotic competence but are of suboptimal quality (PND 16 large), and oocytes that have meiotic competence and are of optimal quality (PND 40). Oocytes were collected from 3 mice per condition to create three independent pools for RNA Seq analysis. All samples had a similar number of read counts and tended to have a higher correlation to samples within the same category as compared to between categories (Supplementary Figures 3A,B). For each condition, the mean RPKM value for each gene was used for further analysis using DESeq2. Differentially expressed genes from pairwise comparisons between conditions were identified based on two criteria: 1.5-fold change and adjusted *P*-value less than or equal to 0.05. Volcano plots demonstrate that the number of differentially expressed genes becomes more pronounced as the difference in animal age increases, with the greatest number of differentially expressed genes observed between oocytes from PND 40 vs. PND 13 mice (Supplementary Figures 4A–C and Table 1). The fewest differentially expressed genes were observed when comparing oocytes from PND 16 small vs. PND 13 mice (Supplementary Figure 4A) and PND 40 vs. PND 16 large mice (Supplementary Figure 4E), suggesting that these groups of oocytes are most similar to each other. Consistent with this, oocytes from PND 16 large and PND 40 mice clustered together relative to PND 13 and PND 16 small mice in a multidimensional scaling (MDS) plot (Figure 6B). Pairwise analysis demonstrated similar expression differences between PND 16 large vs. PND 13, PND 40 vs. PND 13, and PND 40 vs. PND 16 small, revealing the gene expression program for maturation (Figure 6C). Interestingly, there were differentially expressed genes between oocytes from PND 16 large and PND 16 small mice despite the animals being the same age, presumably reflecting the different maturation of these oocytes (Figure 6C and Supplementary Figure 4D).

**TABLE 1:** Pairwise analysis of differentially expressed genes (fold change +1.5 and −1.5 and adj *p* < 0.05) across different oocyte cohorts.

S. No.	Comparison	Number of upregulated genes	Number of downregulated genes
1	PND16 small vs. PND13	57	138
2	PND16 large vs. PND13	504	1,445
3	PND40 vs. PND13	894	1,861
4	PND16 large vs. PND16 small	178	537
5	PND40 vs. PND16 large	511	252
6	PND40 vs. PND16 small	793	1,313
7	PND 16 large and PND 40 vs. PND 13 and PND 16 small	580	1,603

**FIGURE 7:**
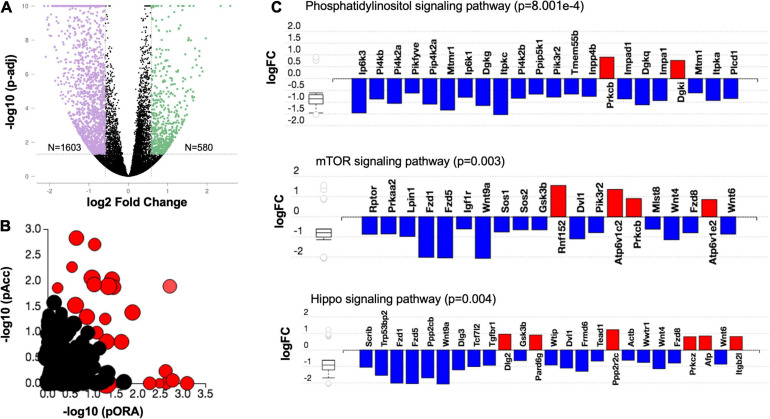
Downregulation of cellular growth pathways in the oocyte is associated with meiotic competence. **(A)** Volcano plot for differentially expressed genes (fold change +1.5 and −1.5 and adjusted *p* < 0.05) between meiotically competent (PND 16 large and PND 40) and non-competent (PND 13 and PND 16 small) oocytes. **(B)** Evidence plot for significantly enriched pathways. Red bubbles represent significant pathways (*p*-value correction factor), and black bubbles represent non-significant pathways. The size of each bubble corresponds to the number of genes affected in that pathway. The horizontal axis shows the inverse log of *p*-value computed using over representation analysis (pORA). The vertical axis shows the inverse log of *p*-value for the accumulated perturbation of the pathway (pAcc). This approach is called the impact analysis and combines the *p*-value into the global *p*-value which is displayed in Table 1. **(C)** Differentially expressed genes in Phosphatidylinositol (*p* = 8.001e–4), mTOR (*p* = 0.003) and Hippo signaling (*p* = 0.004) pathways in meiotically competent as compared to sub-competent oocytes.

To identify gene expression profiles that underlie meiotic competence, we compared differentially expressed genes between meiotically non-competent (PND 13 and PND 16 small) and competent (PND 16 large and PND 40) oocyte cohorts. These populations are in fact significantly different with respect to their gene expression as evidenced by the volcano plot (Figure 7A). Interestingly, when we performed an over representation analysis (ORA), we saw that there is a significant perturbation of several pathways (Figure 7B). Based on Gene Ontology analysis, genes involved in phosphatidylinositide 3-kinase, mTOR, and Hippo signaling pathways were predominantly downregulated in the age cohort associated with oocyte meiotic competence (PND 16 large and PND 40) (Table 2 and Figure 7C).

**TABLE 2:** Top 10 enriched pathways between meiotically competent (PND 16 large and PND 40) and non-competent (PND 16 small and PND 13) oocytes.

Pathway name	*p*-value
Melanogenesis	2.918e–4
Phosphatidylinositol signaling system	8.001e–4
Basal cell carcinoma	0.002
N-Glycan biosynthesis	0.002
mTOR signaling pathway	0.003
Hepatocellular carcinoma	0.003
Glycosaminoglycan biosynthesis—chondroitin sulfate	0.003
Hippo signaling pathway	0.004
Proteoglycans in cancer	0.005
Human papillomavirus infection	0.005

*PND, postnatal day.*

Oocytes from PND 16 large and PND 40 mice are both meiotically competent, but those from PND 40 mice are of better quality based on their follicular origins, spindle morphology, and chromosome configuration. Thus, comparison of gene expression between these cohorts allows interrogation of pathways required for gamete quality. Gene Ontology analysis revealed that differentially expressed genes involved in cellular communication (i.e., cell periphery, plasma membrane, postsynapse, extracellular region, cell junction, extracellular vesicle, dense core granule) are upregulated in oocytes from PND 40 mice (Table 3). Thus, interaction between the oocyte and its surrounding cumulus cells at this stage is fundamental to the development of a high-quality gamete.

**TABLE 3:** Top 10 GO term summary (cellular components) between PND 40 and PND 16 large oocytes.

Name gene	(DE/All)	*p*-value
Cell periphery	216/3,980	3.700e-5
Plasma membrane	209/3,868	7.000e-5
Plasma membrane part	122/2,073	1.300e–4
Dense core granule	5/16	4.100e–4
Postsynapse	34/445	8.000e–4
Extracellular region	189/3,606	9.100e–4
Synapse part	47/685	9.600e–4
Cell junction	72/1,165	9.800e–4
Synapse	56/859	0.001
Extracellular vesicle	132/2,412	0.001

*DE, differential expression, PND, postnatal day.*

## Discussion

Advances in pediatric fertility preservation are necessitating a better understanding of egg quality in young girls. Ovarian tissue cryopreservation (OTC) is the mainstay of fertility preservation for prepubertal females (Practice Committee of the American Society for Reproductive Medicine, 2019). However, live birth rates following ovarian tissue transplantation are low, and in some cases there is a risk of reseeding malignant cells upon transplantation (Meirow et al., 2008; Donnez et al., 2015; Van der Ven et al., 2016). Therefore, isolation of immature oocytes and follicles from ovarian tissues with subsequent *in vitro* growth and maturation is a vigorous research area. There are also emerging reports of ovarian stimulation, oocyte retrieval, and cryopreservation from pre-pubertal females (Reichman et al., 2012; Azem et al., 2020). Thus, understanding the quality of these gametes is of paramount importance especially because studies in multiple animal species and humans have raised concerns regarding the suboptimal quality of gametes in this population (Franasiak et al., 2014; Duncan, 2017; Gruhn et al., 2019).

Here we report the characterization and use of a mouse model of the pubertal transition to understand egg quality during this period. Our mouse model recapitulated key features of puberty in humans, including increase in weight and rising inhibin B levels (Raivio and Dunkel, 2002). As mice progressed through puberty, there was an increase in the ability of oocytes to undergo meiotic maturation, and there may be a potential association with animal weight as well. This is consistent with previous observations which showed that the developmental competence of *in vitro* matured mouse oocytes is higher in 26-day old compared to 22-day old mice (Eppig et al., 1992). We narrowed down PND 16 as an important developmental switch in the acquisition of meiotic competence in the CD-1 mouse strain. This was an unexpected and interesting finding. There were only 2 mice in each group and thus we chose not to show these data in the current manuscript due to small number of animals. However, it is an important and active area of further investigation. Meiotic competence correlated with the increased size of the follicles and oocytes, and this is consistent with previous studies demonstrating that oocyte and follicular size are important factors in determining oocyte developmental competence during the pubertal transition in mice (Eppig and Schroeder, 1989; Eppig et al., 1992). In pigs, increased follicle size is associated with improved meiotic maturation and developmental competence (Marchal et al., 2002). Interestingly, the size-dependence is also observed *in vitro* because there is an association between increasing size of the antral follicle and improved meiotic and embryo developmental competence in isolated mouse follicles grown in alginate hydrogels (Xiao et al., 2015). Consistent with animal studies, data from humans also show that oocyte diameter needs to reach a certain size, 112 μm in one study (Duncan et al., 2012), before meiotic competence is obtained.

Although the ability of an oocyte to reach MII is a prerequisite to form a gamete that can undergo normal fertilization, just because a gamete extrudes a first polar body does not mean it is of high quality. In fact, we observed a higher incidence of chromosome configuration abnormalities in meiotically competent oocytes from mice early during the pubertal transition compared to older age cohorts. These observations are consistent with what is observed in human where there is an increased incidence of aneuploidy in oocytes and embryos obtained from girls during the pre-pubertal period and in early years of adolescence and the reproductive lifespan (Franasiak et al., 2014; Gruhn et al., 2019). Although chromosome abnormalities are common at both ends of the age spectrum, there are differences in the phenotypes suggesting distinct underlying mechanisms. For example, in the mouse model, our data suggests that cell cycle delays and telophase configurations are predominant abnormalities in oocytes from mice during the pubertal transition. In contrast, loss of chromosome cohesion and premature separation of sister chromatids (PSSC) is the predominant mechanism of aneuploidy associated with advanced reproductive age (Chiang et al., 2010). In humans, meiotic non-disjunction appears to be the predominant mechanism responsible for aneuploidy in women <20 years old compared to PSSC or reverse segregation which increases with advanced age and is the primary mechanism of aneuploidy in older women (Duncan et al., 2012; Gruhn et al., 2019). Thus, while aneuploidy associated with advanced reproductive age appears to be due to a deterioration of pathways related to chromosome segregation (Pan et al., 2008; Duncan et al., 2017), the defects observed in oocytes in juvenescence are likely due to the immature status of the gametes.

In our study, the acquisition of meiotic competence during the pubertal transition was associated with decreased expression of cellular growth pathways, including PI3K, mTOR, and Hippo. The differences in these transcriptomic signatures could be secondary to the antral follicle size differences between age groups, which is not surprising given immaturity of hypothalamic-pituitary-ovarian (HPO) axis in pre-pubertal animals. Although there are many reports highlighting the importance of these signaling pathways in primordial follicle activation (John et al., 2008; Reddy et al., 2008; Kawamura et al., 2013; Sun et al., 2015; Yan et al., 2018), studies examining their role in continued oocyte growth and meiotic maturation are limited. In one study, conditional knockout of mTOR in growing oocytes resulted in severely compromised oocyte developmental competence despite only slight reduction in the number of ovulated oocytes (Guo et al., 2018). Interestingly, the majority of these oocytes demonstrated chromosome configuration defects in meiosis I where telophase I configuration was the predominant abnormality. These findings along with the results of our study point to the role of these growth pathways beyond early folliculogenesis.

In many cells, there is a direct relationship between cell size and cell cycle (Goranov et al., 2009), with growth and cell cycle progression being highly intertwined (Jorgensen and Tyers, 2004). Inhibition of cell growth leads to cell cycle arrest (Temin, 1971; Pardee, 1974; Prescott, 1976; Jorgensen and Tyers, 2004) and activation of growth pathways drives cell cycle progression (Backman et al., 2002; Rupes, 2002; Saucedo and Edgar, 2002). Mitotic cells enforce critical cell size thresholds at G1/S and/or G2/M phase transitions (Shields et al., 1978; Dolznig et al., 2004; Jorgensen and Tyers, 2004). Similar mechanisms might be at play in oocytes. Oocyte growth is associated with the accumulation of cell cycle proteins including Cyclin B, p34^*CDC*2^ and CDC25 (Chesnel and Eppig, 1995; Kanatsu-Shinohara et al., 2000), and the threshold amount of these proteins is thought to be needed for meiotic progression. There is also an association between the oocyte size and meiotic competence (Duncan et al., 2012). Thus, the observed association of decreased expression of genes involved in growth pathways with oocyte meiotic competence may reflect that this population of oocytes has reached a critical size threshold and has accumulated the necessary molecules to undergo meiosis. Therefore, these growth pathways are downregulated as the oocyte diverts resources from growth to support downstream processes of meiotic maturation, fertilization, and preimplantation embryo development.

Although oocyte meiotic progression was similar across ages following PND 16, the quality of the gametes was not because there were fewer chromosome abnormalities in oocytes from the older age cohorts. There appears to be fundamental biological differences in follicles that occur with age because even in human, the same stage follicles obtained from pre-pubertal girls show compromised *in vitro* growth potential compared to those isolated from adults (Anderson et al., 2014). In our mouse model, we demonstrated that oocytes from younger mice come from smaller antral follicles with fewer cumulus cell layers relative to the older mice. Moreover, genes that regulate cellular communication are differentially expressed when comparing oocytes from PND 16 large and PND 40 cohorts even though oocytes from both populations of mice are meiotically competent. This highlights the potential mechanism of quality difference between gametes obtained during the pubertal transition and from adults.

Communication between the oocyte and cumulus-granulosa cells is pivotal for oocyte growth, metabolism, and maintenance of meiotic arrest (Ackert et al., 2001; Eppig et al., 2005; Sugiura et al., 2005; Su et al., 2008; Kidder and Vanderhyden, 2010; Wigglesworth et al., 2013; Dumesic et al., 2015; El-Hayek and Clarke, 2015; El-Hayek et al., 2018). Despite having comparably high meiotic maturation rate, oocytes with thicker cumulus cell layers appear to have higher blastocyst development rate in mice (Zhou et al., 2014). We also noted that the ZP was thinner in younger mice and increased throughout the pubertal transition. The ZP is important for mediating bi-directional communication between the oocyte and its surrounding granulosa cells via transzonal projections (TZPs) and associated gap junctions. The increase in zona thickness and increased expression of cellular communication genes that occurs coincidently with animal age across the pubertal transition underscores the potential important role of transzonal projections (TZPs) in determining gamete quality during this period. Thus, impaired bidirectional communication between the oocyte and granulosa cells may be shared mechanisms of reduced gamete quality at both ends of the aging spectrum, since TZP number and function is decreased in reproductively old mice (El-Hayek et al., 2018).

In summary, our work demonstrates that the pubertal transition in female mice is associated with suboptimal gamete quality. In juvenescence, insufficiently activated growth pathways or cellular communication with surrounding somatic cells appear to be the predominant mechanisms responsible for poor gamete quality. This is in contrast to reproductively old mice where advanced age is associated with perturbation of degradation of mRNAs and altered expression of genes important in spindle formation, kinetochore-microtubule attachment, and chromosome segregation (Pan et al., 2008). Our findings have important translational implications. For example, age and/or weight of the pediatric patient undergoing fertility preservation may have prognostic value for future reproduction that may help clinicians better counsel patients and develop appropriate treatment plans. In addition, the non-invasive assessment of meiotic spindles using the PolScope (Moon et al., 2003; Konc et al., 2004; Rama Raju et al., 2007) may aid in selection through distinguishing morphologically mature eggs with polar bodies that have cell cycle abnormalities (i.e., telophase I). We can also use our results to improve clinical IVM strategies. C-type natriuretic peptide (CNP) pretreatment of immature oocytes from PCOS patients before IVM was previously shown to improve maturation rates and embryo development (Sanchez et al., 2019). CNP maintains the oocytes in a protracted period of meiotic arrest via activation of the NPR2 receptor (Gilchrist et al., 2016; Sugimura et al., 2018) and likely improves cytoplasmic maturation by allowing more time for the accumulation of factors necessary to support meiosis and early embryo development. However, stimulation of growth pathways in addition to the maintenance of meiotic arrest may be necessary to further improve IVM outcomes, and this can be combined with the development of oocyte diameter thresholds to assess meiotic competency. Moreover, the elucidation of molecular mechanisms regulating the switch from oocyte growth to the acquisition of meiotic competence and investigating whether there is an active mechanism of suppression of these growth pathways will provide important clues for our understanding of oocyte maturation.

## Materials and Methods

### Animals

Reproductively adult CD-1 male and female mice were obtained from Envigo (Indianapolis, IN) and used to establish an in-house breeding colony. Mice were maintained in a temperature-, humidity-, and light- (14 h light:10 h darkness) controlled barrier facility within Northwestern University’s Center of Comparative Medicine. Mice were provided with food and water *ad libitum*. Diet formulated to exclude soybean meal, which minimizes the presence of isoflavones, the primary type of phytoestrogen was used (Teklad Global irradiated 2916 chow: Envigo, Madison, WI). CD-1 female pups in the following aged cohorts that spanned the pubertal transition were obtained from the breeding colony: postnatal day (PND) 11–15, 16–20, 21–25, 26–30, 31–35, and 36–40. A total of 58 mice were used in this study, with between 7 and 15 mice used in each age cohort. Prior to euthanasia, mice were weighed, and the pubertal status of each animal was verified by visual analysis of the vaginal opening as described previously (McLean et al., 2012). All experiments were performed in accordance with the National Institutes of Health Guidelines for Care and Use of Laboratory Animals and under protocols approved by the Northwestern University Institutional Animal Care and Use Committee (IACUC).

### Hormone Assays

Serum estradiol (E2) and inhibin B levels were assessed by the University of Virginia’s Center for Research in Reproduction—Ligand Assay and Analysis Core. All blood samples were drawn from the inferior vena cava, clotted, and centrifuged. Serum was stored at −80°C until hormone analyses. Estradiol concentrations were determined using Mouse/Rat Estradiol ELISA kit (Calbiotech, El Cajon, CA), and the intraassay coefficient of variation (CV) was 6.8% and the interassay CV was 8.9%. Inhibin B concentrations were determined using an Inhibin B (ELISA) kit (AnschLabs, Webster, TX). The intraassay CV was 4.8%, and the interassay CV was 6.7%. Samples and standards for each gonadal hormone assay were run in duplicate. Blood was taken from between 3 and 5 mice in each age cohort. Samples were run for individual animals except in the PND 11–15 cohort where one pooled sample from 5 mice was run due to insufficient volume of blood that can be collected from animals at these young ages.

### Oocyte Collection and Imaging

Ovaries were harvested from unstimulated mice (no exposure to gonadotropins) to mimic *ex vivo* IVM in the fertility preservation setting. Cumulus oocyte complexes (COCs) were isolated from the ovaries by poking antral follicles using 25-gauge needles. Collection media was Leibovitz’s L-15 medium (Life Technologies, Grand Island, NY, United States) supplemented with 3 mg/ml polyvinylpyrrolidone (PVP) (Sigma-Aldrich Co., St. Louis, MO, United States) and 0.3% penicillin-streptomycin (PS) (Life Technologies, Grand Island, NY, United States). Ten micrometer milrinone was also added to the media to prevent spontaneous meiotic resumption which is an oocyte-specific specific phosphodiesterase 3 inhibitor that maintains meiotic arrest by inhibiting cAMP degradation (Jensen et al., 2002). Cumulus cells were mechanically removed from the oocytes by gentle pipetting, and denuded oocytes containing intact germinal vesicles (GV) were transferred into media consisting of α-MEM Glutamax (MEM) (Life Technologies, Grand Island, NY, United States) supplemented with 3 mg/ml Bovine Serum Albumin (BSA), 0.5% PS (MEM/BSA/PS), and 10 μM milrinone for short-term culture in a humidified atmosphere of 5% CO_2_ in air at 37°C. Oocytes were imaged by brightfield microscopy using a 20x objective on an EVOS FL Auto live imaging microscope system (Life Technologies, Grand Island, NY, United States). Average oocyte diameter measurements were made using the EVOS FL Auto based on two perpendicular measurements from plasma membrane to plasma membrane of each oocyte. The thickness of the zona pellucida was also measured on images taken on the EVOS FL Auto.

### *In vitro* Maturation and Scoring of Meiotic Progression

To initiate synchronous and spontaneous meiotic resumption, oocytes were rinsed through large droplets of L15/PVP/PS to remove milrinone and placed in 1 ml of MEM/BSA/PS. Oocytes were *in vitro* matured for 14–16 h in a humidified atmosphere of 5% CO_2_ in air at 37°C. Following IVM, oocytes were evaluated by brightfield microscopy, and their meiotic stage was assessed using established morphological criteria (Treff et al., 2016). In brief, oocytes with a visible nucleus were arrested at prophase of meiosis I in the germinal vesicle (GV)-intact stage. Oocytes without a nucleus or a polar body were considered to have undergone germinal vesicle breakdown (GVBD) or were at metaphase of meiosis I (MI). If polar bodies were visible in the perivitelline space, the oocytes were considered arrested at metaphase of meiosis II (MII). Fragmented, shrunken, or misshapen oocytes were classified as degenerate (D).

### Immunofluorescence, Confocal Microscopy, and Spindle Assessment

To assess spindle morphology, oocytes were fixed in 3.8% paraformaldehyde (Electron Microscopy Science Hatfield, PA) in phosphate buffered saline (PBS) containing 0.1% Triton-X-100 for 1 h at 37°C. After washing three times in blocking buffer containing phosphate buffered saline (PBS) containing 0.3% albumin (BSA), 0.01% Tween-20, and 0.02% sodium azide (NaN_3_), oocytes were then permeabilized in PBS containing 0.3% BSA, 0.1% Triton-X-100, and 0.02% NaN_3_ for 15 min at room temperature. Samples were then washed twice in blocking buffer and microtubules were stained with an alpha tubulin 488-conjugated anti-rabbit primary antibody (11H10; Cell Signaling Technology, Danvers, MA) (1:100 dilution) for 2 h at room temperature. Oocytes were rinsed three times in blocking buffer and then mounted on glass slides in Vectashield containing 4′,6-diamidino-2-phenylindole (DAPI; Vector Laboratories, Burlingame, CA). Oocytes were imaged on a Leica SP5 inverted laser scanning confocal microscope (Leica Microsystems) using a 63X objective and the 405 and 488 nm lasers. For each oocyte, 0.5 μm optical thick sections were taken through the region of the spindle, and stacks or maximum projections were analyzed. Spindle length was measured from pole-to-pole based on tubulin fluorescence using Image J software (National Institutes of Health, Bethesda, MD). Spindles that were oriented perpendicular to the image plane were excluded from the analysis. Chromosome configuration was analyzed and categorized as follows: normal (no misaligned chromosomes), 1 misaligned chromosome on the metaphase plate, >1 misaligned chromosome on the metaphase plate, and other. The other category included cells that were at the incorrect cell cycle stage such as anaphase or telophase.

### Histological Analysis

Ovaries were harvested from mice across age cohorts, and the bursa and excess fat were removed. Ovaries were fixed in Modified Davidson’s fixative, a combination of 14% ethyl alcohol, 37.5% formalin, 37–39% glacial acetic acid in deionized water (Electron Microscopy Sciences, Hatfield, PA). Ovaries were fixed at room temperature for 6 h with gentle agitation and then transferred to 4°C overnight (∼15 h). Samples were washed and stored in 70% ethanol. Each ovary was processed and dehydrated using an automated tissue processor (Leica Biosystems, Buffalo Grove, IL) and embedded in paraffin wax (Leica EG1160, Leica Biosystems). Ovaries were serial sectioned at a 5 μm thickness, and every fifth section of a ribbon was placed on a separate slide designated for Hematoxylin and Eosin (H&E) staining. Slides were H&E stained using the AutostainerXL (Leica Biosystems) and then imaged using the EVOS FLUO Auto microscope. All sections were examined. Average follicle and corresponding diameter measurements were made within histological images using the EVOS FL Auto software. Only follicles with an oocyte nucleus visible in the center were used for measurements as this represented the mid-point of the follicle. Follicle measurements were based on two perpendicular measurements from basement membrane-to-basement membrane, and oocyte measurements were based off of two perpendicular measurements from plasma membrane-to-plasma membrane. The number of cumulus cell layers surrounding each oocyte in antral follicles were also counted.

### RNASeq in Oocytes

Oocytes were collected from four cohorts of mice: PND 13, PND 16 small (mice < 9 g), PND 16 large (mice ≥ 9 g), and PND 40 mice. For each cohort, pooled oocytes were snap frozen in three replicates. Each replicate had 18–45 oocytes. RNA was isolated using RNeasy Plus mini kit (Qiagen, Germany, 74134). cDNA synthesis was performed using NEB Next Ultra II directional RNA library prep kit (New England Biolabs, Ipswich, MA, E7760S) after rRNA depletion (NEB Next rRNA depletion kit, E6310S) for Illumina next generation sequencing (HiSeq-50 bp). Data were analyzed by Rosalind^1^, with a Hyper Scale architecture developed by OnRamp BioInformatics, Inc. (San Diego, CA). Additional gene enrichment is available from the following partner institutions: Advaita^2^.

### Statistical Analysis

Statistical analysis was performed using GraphPad Prism software (version 9.0.1 for Mac, GraphPad Software, San Diego, California United States). Categorical and numerical variables were analyzed using one-way ANOVA, Kruskal-Wallis, *t*-test, chi-square, Fisher’s exact or Spearmen correlation tests where relevant following normality tests where necessary. *P* < 0.05 was considered statistically significant. Tukey test was used for multiple comparisons following one-way ANOVA.

## Data Availability Statement

The data was deposited in Gene Expression Omnibus—GEO accession number is GSE171858 (https://www.ncbi.nlm.nih.gov/geo/query/acc.cgi?acc=GSE171858). Original data underlying this manuscript can also be accessed from the Stowers Original Data Repository at http://www.stowers.org/research/publications/libpb-1617.

## Ethics Statement

The animal study was reviewed and approved by the Northwestern University Institutional Animal Care and Use Committee.

## Author Contributions

FD, JG, and AK: conceptualization, funding, resources, supervision, and methodology. FD: project administration. AK, VS, FD, and EB: writing. All authors: investigation, formal analysis, validation, visualization, and final approval.

## Conflict of Interest

The authors declare that the research was conducted in the absence of any commercial or financial relationships that could be construed as a potential conflict of interest.

## Footnotes

1 https://rosalind.onramp.bio/

2 http://www.advaitabio.com/ipathwayguide
