# Supplementary figures and images for: Immature Follicular Origins and Disrupted Oocyte Growth Pathways Contribute to Decreased Gamete Quality During Reproductive Juvenescence in Mice
Source: Front Cell Dev Biol. 2021 Jun 16;9:693742. doi: 10.3389/fcell.2021.693742 (PMC8244820; doi:10.3389/fcell.2021.693742)

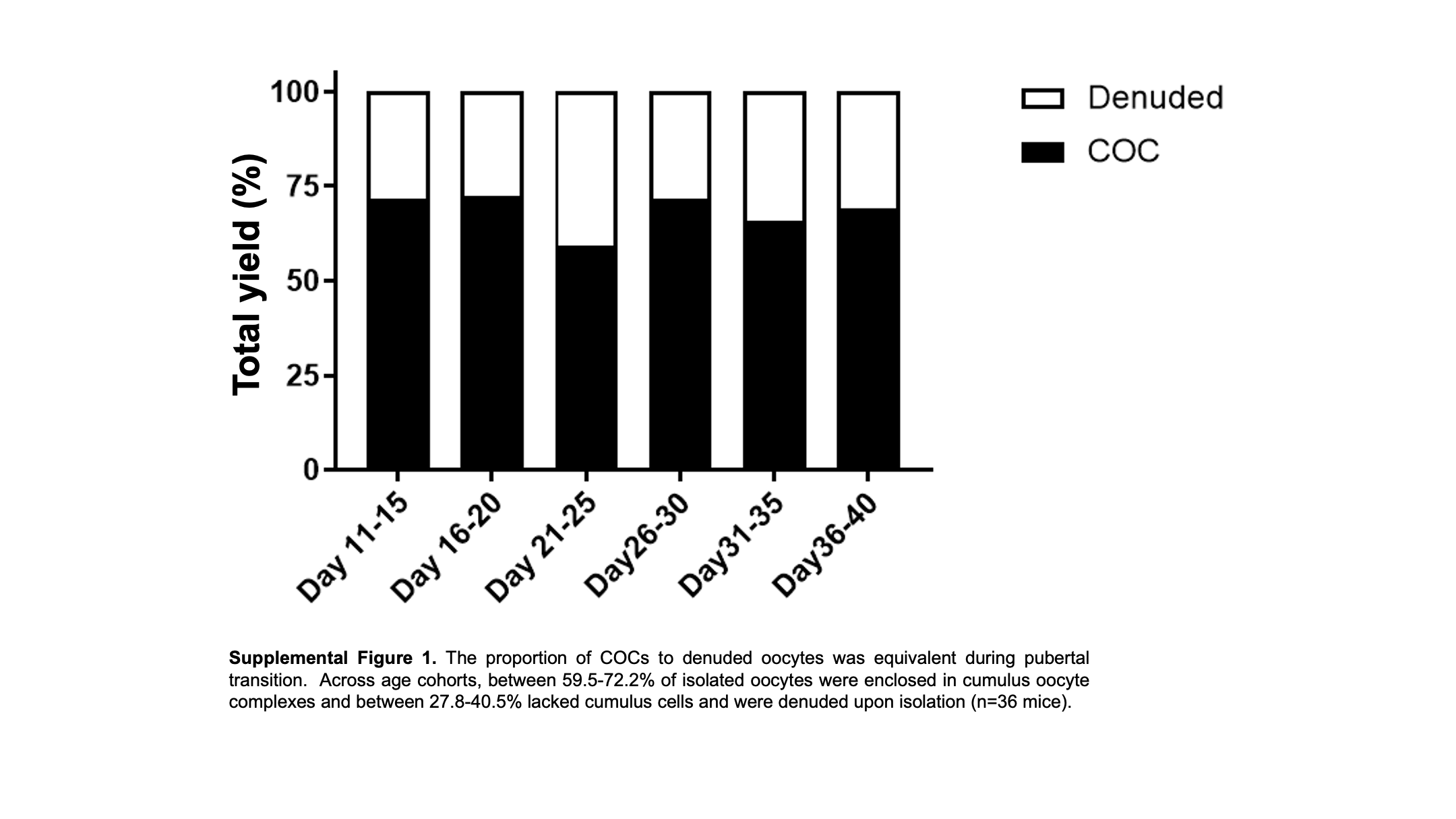

Supplement: Supplementary file 1 [file Image_1.TIFF]

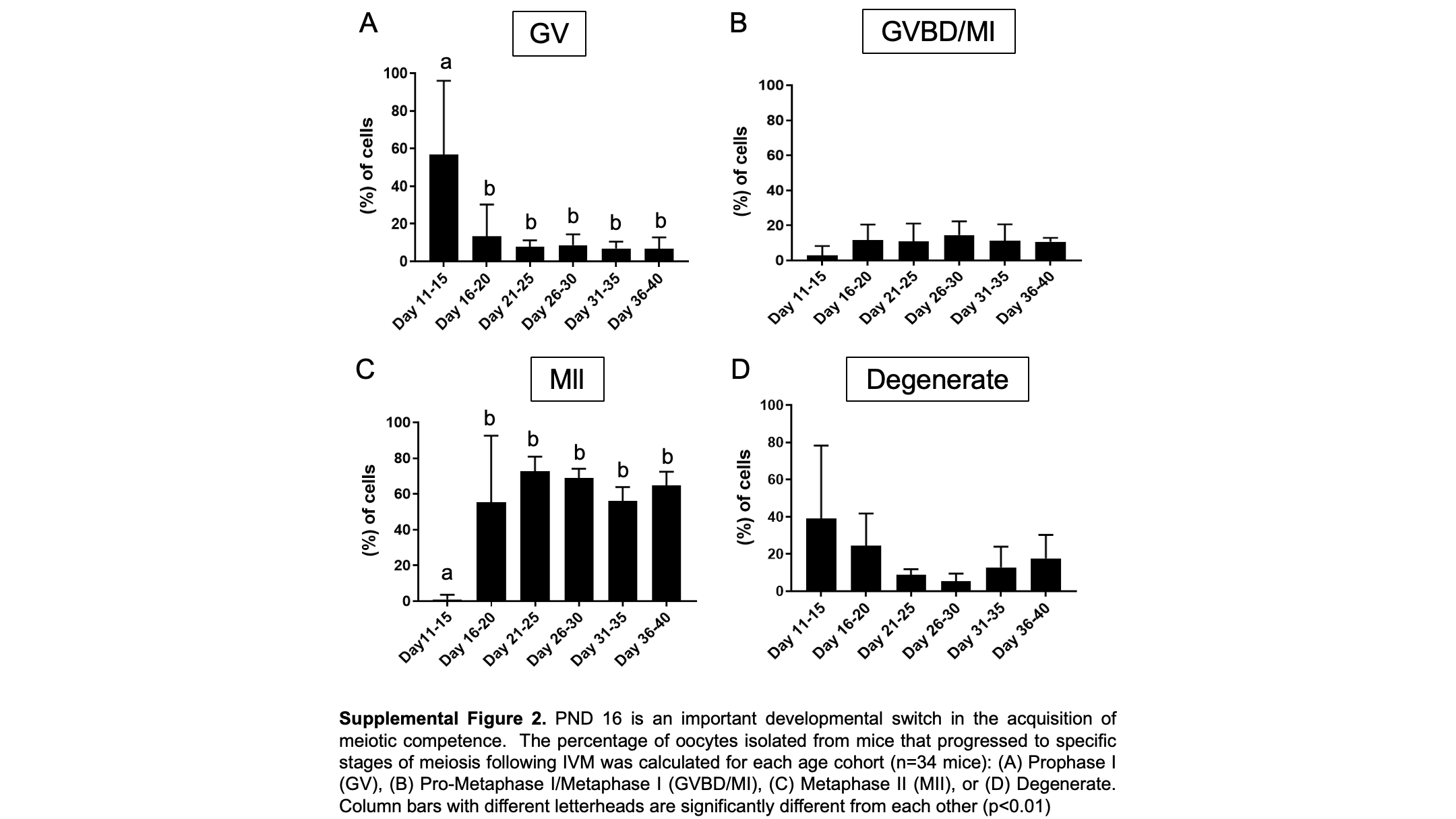

Supplement: Supplementary file 2 [file Image_2.TIFF]

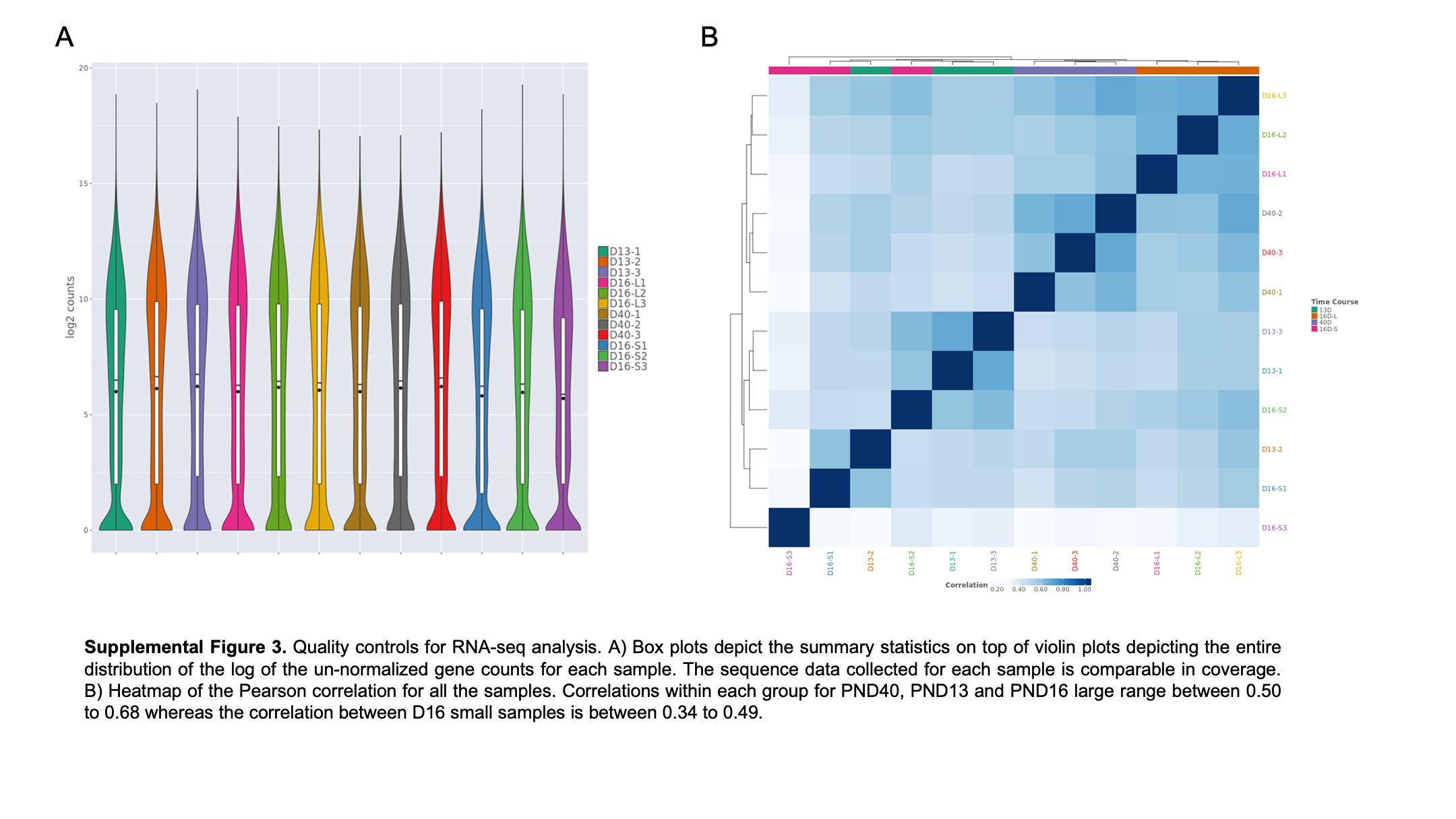

Supplement: Supplementary file 3 [file Image_3.TIFF]

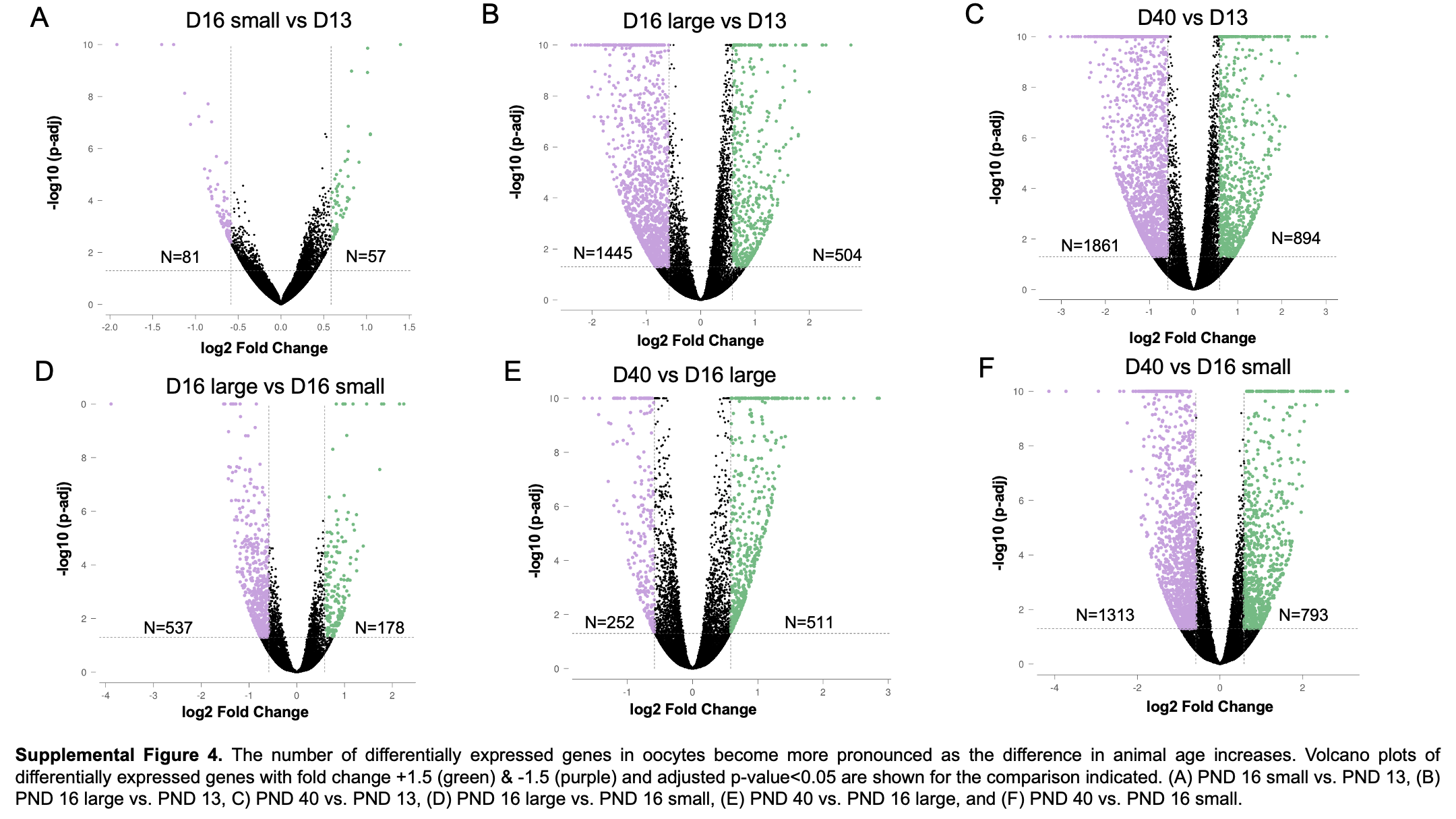

Supplement: Supplementary file 4 [file Image_4.TIFF]
